# Cysteine-rich intestinal protein family: structural overview, functional diversity, and roles in human disease

**DOI:** 10.1038/s41420-025-02395-y

**Published:** 2025-03-21

**Authors:** Xilin Ye, Qianben Song, Lumiao Zhang, Mengjia Jing, Yu Fu, Wei Yan

**Affiliations:** 1 Department of Gastroenterology, Tongji Hospital, Tongji Medical College, Huazhong University of Science and Technology, Wuhan, China; 2 Department of Gastroenterology, Union Hospital, Tongji Medical College, Huazhong University of Science and Technology, Wuhan, China

**Keywords:** Metastasis, Tumour biomarkers

## Abstract

The cysteine-rich intestinal protein (CRIP) family, including CRIP1, CRIP2, and CRIP3, is a subfamily of the highly conserved Lin-1, Isl1, Mec3/double zinc finger protein family that exhibits diverse biological functions. The CRIP family is known to play an important role in cellular epithelial-mesenchymal transition, cell death, and tumor progression and participate in multiple signaling pathways. This article summarizes the roles and potential molecular mechanisms of the CRIP family in diseases, which will help to explore new research directions for this family and provide useful information for clinical applications such as disease diagnosis and treatment.

## Facts

- The CRIP family includes CRIP1, CRIP2, and CRIP3, which are low molecular weight zinc-binding proteins that contain one or two conserved LIM domains.
- Different members of the CRIP family play various roles in the same tumor type, and the action of CRIP1 in different tumors is related to the activation of multiple signaling pathways.
- The clinical development of CRIP2 inhibitors could provide a targeted therapeutic approach for patients with tumors to overcome apoptosis resistance and improve therapeutic efficacy.
- The CRIP family is effective in predicting cardiovascular disease risk and is expected to become an important link in the treatment of cardiovascular disease.

## Open questions

- The underlying mechanisms by which the CRIP family regulates disease and cancer remain largely obscure.
- The interactions and regulation between CRIP family members are unknown.
- Molecular inhibitors targeting the CRIP family are yet to be developed.

## Introduction

With the development of genomics and proteomics technology, more protein families have been found to be closely related to a variety of biological processes. The cysteine-rich intestinal protein family (CRIP), also known as cysteine-rich protein, is a subfamily of the Lin-1, Isl1, Mec3 (LIM) protein/double zinc finger protein family, consisting of three family members: CRIP1, CRIP2, and CRIP3. The CRIP family proteins are low molecular weight zinc-binding proteins containing one or two conserved LIM domains [1]. The LIM domain is a double zinc finger motif composed of 50–65 amino acids, characterized by a high concentration of seven cysteine residues and one histidine residue, capable of binding two zinc ions to maintain its stable structure [2]. However, in contrast to the Gada-like zinc finger structure, the LIM domain is specifically responsible for protein interactions and not involved in DNA binding. LIM domains are involved in intracellular signal transduction through protein-protein interactions and interact with co-activators, co-repressors, competing factors, and other transcription factors to regulate gene expression [2], thus playing a key role in embryonic and nervous system development [3]. Owing to the biological functions of the LIM domain, CRIP is thought to play an essential role in eukaryotic growth and differentiation, and its expression can be induced by zinc, cytokines, and hormones [4–6].

CRIP1 was first discovered in 1986 by Birkenmeier et al. while screening small intestinal cDNA libraries of rats and mice [7]. Since then, the structure and biological functions of CRIP family members have been continuously elucidated. Studies of the CRIP family initially emphasized its role in intestinal zinc absorption, and Hempe et al. showed that CRIP1 competitively binds zinc with metallothionein during intestinal zinc absorption which is related to the functional diversity of LIM domains [8]. In addition, some scholars have speculated that CRIP is a mammalian member of the ancient ferredoxin superfamily, suggesting that CRIP1 may bind to iron and participate in electron transport and iron metabolism [7], whereas CRIP2 may bind to copper to regulate specific cellular activities [9]. Although the coordination of CRIP3 with metal ions has not been fully revealed, the binding of the LIM domain to the metal is unquestionable. The coordination of the CRIP family proteins and metal ions makes them a key factor in regulating cell functions, maintaining the stability of intracellular and extracellular environments, and participating in various biological processes.

In addition to their fundamental roles in normal cell function, CRIP members are thought to play important roles in the regulation related to differentiation [9], angiogenesis [10], immune function [11], and carcinogenesis [12] and are closely associated with the overall survival and prognosis of patients with cancer, including breast [13] and gastric cancers [10]. In addition, aberrant CRIP protein expression has been observed in other diseases, such as fibrotic [14], cardiovascular [15], and neurological diseases [16]. Although advances have been made in CRIP research in recent years, the specific mechanisms of the CRIP family in a variety of biological processes and disease progression remain unclear; and the functional differences among the CRIP family members and their roles in different tissues have not been fully elucidated. Therefore, this review summarizes the functional roles of the CRIP family in various biological processes and disease development, its dual function as a tumor suppressor gene or oncogene, its interaction with other proteins, and the clinical significance of targeted therapy in cancer progression, to provide effective ways for clinical disease diagnosis and potential therapeutic targets.

## Overview of the CRIP family: domain, expression, and research history

CRIP1(Gene ID: 1396 Uniprot ID: P50238), also known as CRP1 and CRHP, is a zinc-binding protein with a single LIM domain [17]. To date, the structure of the rat CRIP1, a peptide with a molecular weight of about 8.5 kDa containing 77 amino acids, has been studied in some detail [18]. In 1996, Psamrez-alvarado et al. determined the structure of CRIP1 using nuclear magnetic resonance spectroscopy, which improved the understanding of the structure of the CRIP family and also revealed its metal-binding properties. CRIP1 binds zinc to form an N-terminal *CCHC* and a C-terminal *CCCC* module. The antiparallel β fold formed by these two modules is closely linked to the C-terminal α-helix and constitutes the three-dimensional structure of CRIP1 [19]. The structural region of the *CRIP1* gene is composed of five exons, and the two zinc finger motifs of its LIM domain are present in the first three exons. Levenson et al. found that the *CRIP1* promoter lacks the classical transcription initiation element, but contains a *GC*-rich region [20]. This enrichment is associated with the presence of *CpG* islands, suggesting that this region may play a key role in transcriptional activity and is susceptible to methylation regulation. Initial data are provided for further exploration of the regulation of *CRIP1* promoter methylation/demethylation status in diseases. In addition, the *CRIP1* gene also has many cytokine signaling elements and glucocorticoid response elements, suggesting that CRIP1 can be regulated by cytokines and hormones [21]. CRIP1 is widely expressed in adult mammals, with high expressions in the small intestine and lung, but is rarely detected in the liver and pancreas [22].

CRIP2(Gene ID: 1397 Uniprot ID: P52943), also known as CRP2, ESP1, and cardiac LIM protein is a previously unrecognized nuclear copper-binding protein identified by Chen et al. [23]. The gene is mapped to human chromosome 14 and encodes 208 amino acids with a predicted molecular weight of 23 kDa [24]. CRIP2 contains two LIM domains that form a paired zinc finger structure separated by two amino acid linkers (*CXXCX17 HXXC*) XX (*CXXCX17 CHXXC*) [25]. The human CRIP2 and rat CRIP1 share most amino acid residues, suggesting that they may exhibit similar cellular functions. CRIP2 had the highest expression level in the heart, moderate expression in the lung, brain, placenta, spleen, and kidney, and low expression in other tissues and organs.

CRIP3(Gene ID: 401262 Uniprot ID: Q6Q6R5) belongs to the same family as CRIP1 and CRIP2. It is also known as CRP3 and thymic LIM protein (TLP) and consists of two LIM structural domains, but its gene is located in human chromosome 6. There are fewer reports and studies on CRIP3. In 2001, Kirchner et al. identified the *CRIP3* gene encoding a TLP which is specifically expressed in the thymus [26]. TLP has a molecular weight of approximately 23 kDa and exists in two isoforms, TLP-A and TLP-B, generated through alternative splicing. These isoforms consist of 204 and 205 amino acids, respectively. Targeted destruction of the TLP can reduce thymocytes but has no significant effect on the positive or negative selection of T-cells [26], suggesting that CRIP3 may play a role in normal thymus development but the regulation of its expression may be complex and unnecessary for T-cell selection.

**Fig. 1:**
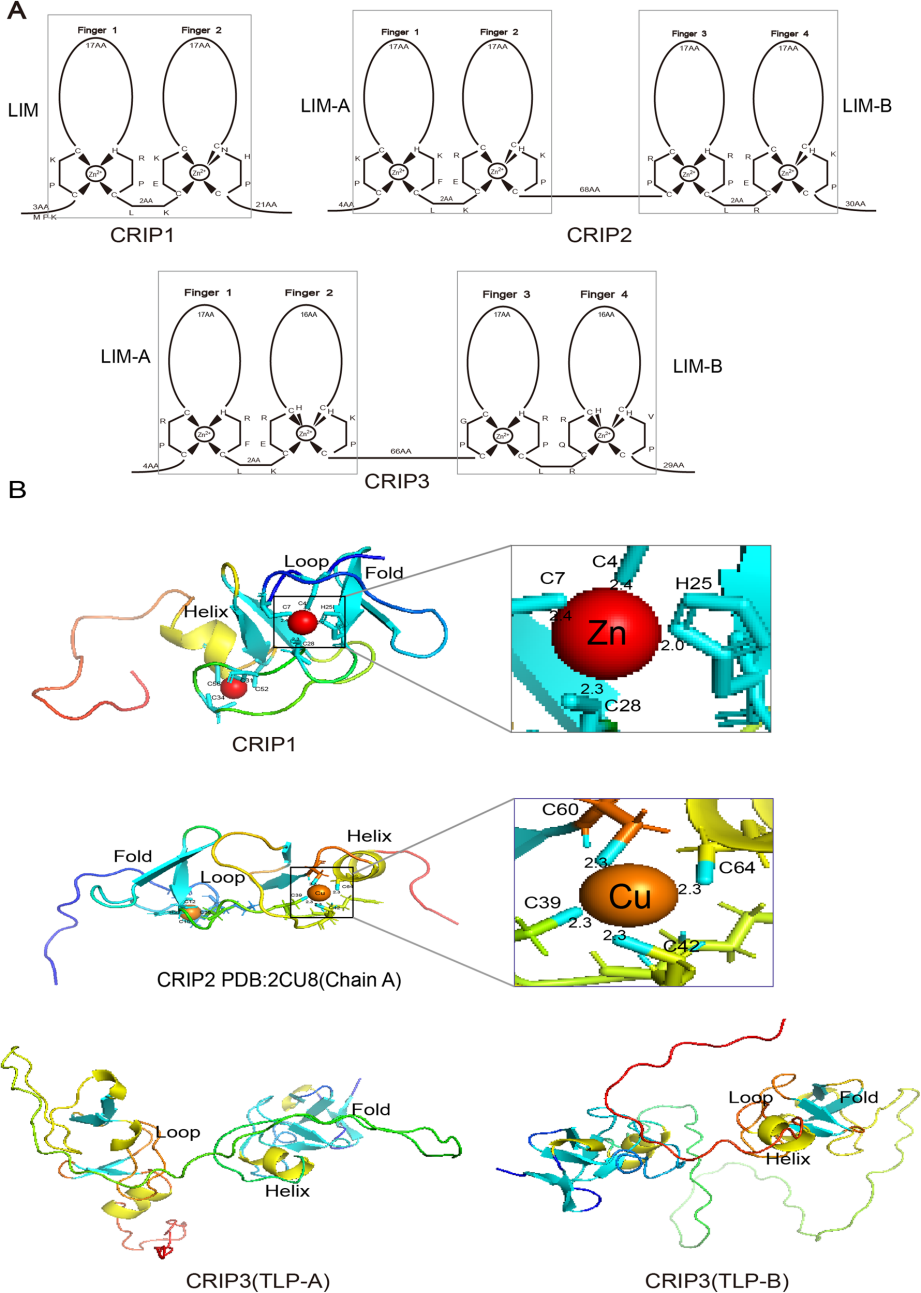
Zinc finger structure and crystal structure of CRIP family. **A** Zinc finger structure of the CRIP family. CRIP1 contains a single LIM domain (comprising two zinc fingers), while CRIP2 and CRIP3 contain two LIM domains (totaling four zinc fingers per protein). **B** Crystal structure of the CRIP family. Top: CRIP1 homology model predicted by the Swiss Model demonstrates zinc-binding coordination geometry, the coordination residues are highlighted. Middle: Chain A of CRIP2 (PDB: 2CU8) in copper-bound conformation, with Cu^+^ coordination residues indicated. Bottom: Two alternatively spliced isoforms of CRIP3 predicted by Swiss-Model. Secondary structure elements are color-coded as follows: • α-helix: yellow cylinders. • β-fold: cyan arrows. • Loop: iridescent lines (connecting ordered structural elements). • Metal coordination spheres: Zn^2+^ (red), Cu^+^ (orange).

In order to further understand the domain and crystal structure of the CRIP family, we used a figure to visually demonstrate the zinc finger structure and three-dimensional conformation of the proteins (Fig. 1).

**Fig. 2:**
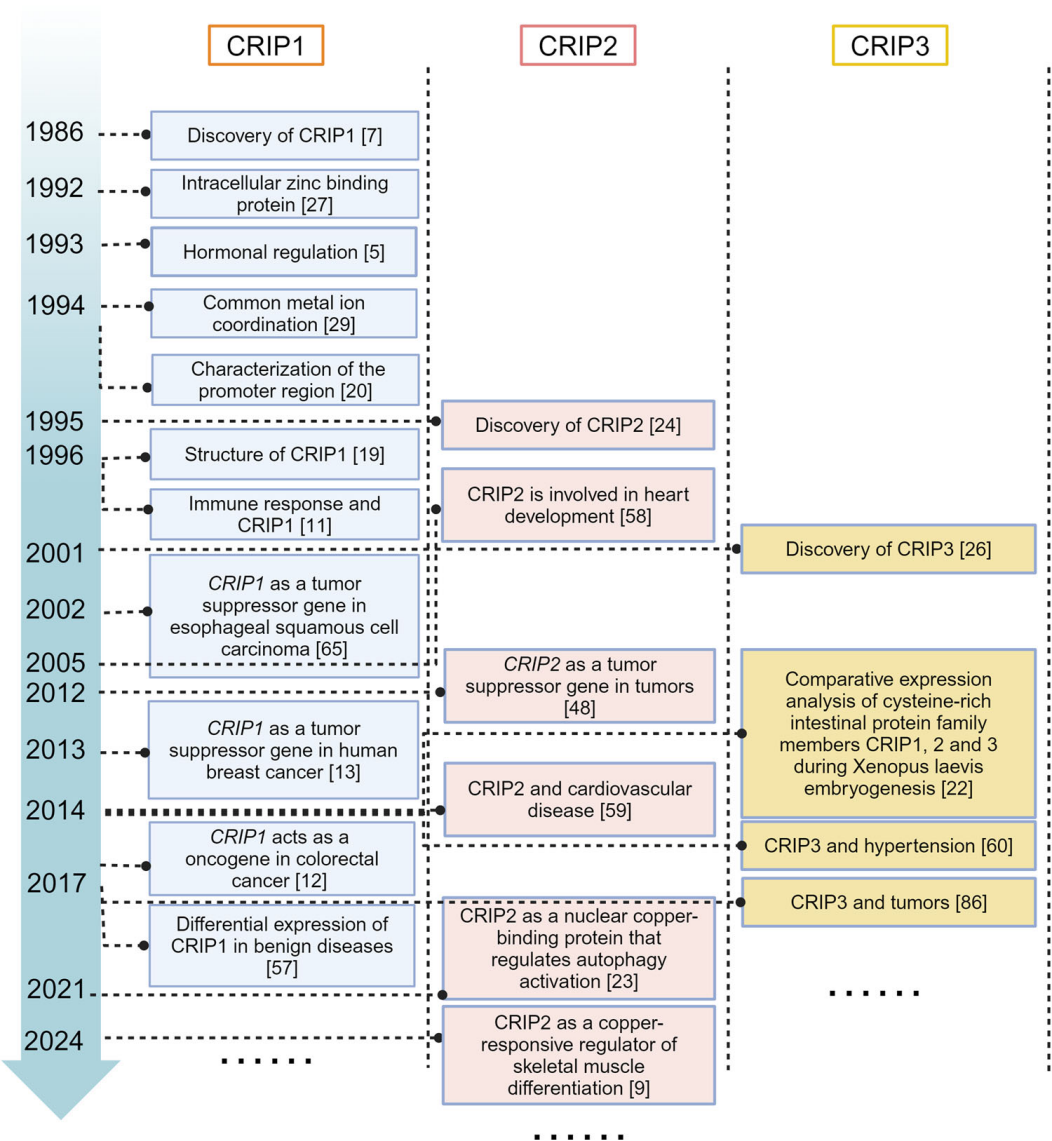
Timeline for CRIP family research history. Spanning from 1986 to 2024, this timeline highlights pivotal discoveries in the CRIP(Cysteine-Rich Intestinal Protein) family, including CRIP1, CRIP2, and CRIP3. The numbers indicate the corresponding reference.

Historically, In 1986, Birkenmeier et al. first discovered the CRIP family when studying the development of the rat gastrointestinal tract, revealing its homology with some ferredoxin proteins [7]. In 1992, Hempe and colleagues proposed a conceptual model of zinc absorption in rats based on the function of CRIP and intestinal metallothionein [27], metallothionein in the model regulates zinc absorption by competitively binding zinc to prevent zinc from binding to CRIP during transcellular diffusion. The results showed that compared with the low-zinc diet, the high-zinc diet significantly increased the concentration of metallothionein, increased the binding ratio of metallothionein to zinc, and decreased the binding ratio of CRIP to zinc, thus affecting the absorption of zinc. This study revealed an important finding that CRIP proteins dynamically bind metal ions through LIM domains, but the molecular pathway by which CRIP promotes zinc absorption in the low-zinc state was not clear because it does not contain a transmembrane domain. Subsequently, CRIP2 was identified as a new member of the CRIP family in 1995 [24], and CRIP3 was discovered in 2001 [26]. It was not until 2014 that Hempel’s team systematically analyzed the spatiotemporal expression characteristics of CRIP family members through the Xenopus laevis embryonic development model. Studies have shown that CRIP1 can be specifically localized to cranial ganglia and neural tube by in situ hybridization at specific embryonic stages (e.g., neurula). CRIP2 showed significant expression signals in the cardiovascular system, brain regions (forebrain and midbrain), and neural tube. The expression profile of CRIP3 further revealed its specific distribution in cranial ganglia and heart tissues [22]. These findings suggest that CRIP family members may be involved in early vertebrate organogenesis through tissue-specific expression patterns. CRIP-based phylogenetic reconstruction and motif analysis suggests the presence of CRIP proteins in the common ancestor of vertebrates, possibly similar to CRIP1; followed by a duplication event that led to the emergence of double-LIM domain protein CRIP2/CRIP3 and the specificity of CRIP2/CRIP3 in vertebrates [9]. These findings establish the phylogenetic importance of the CRIP family, and the different tissue distributions of CRIP families suggest that they may have unique but overlapping functions. At the same time, studies have found that CRIP1 and CRIP2 are regulated by hormones such as glucocorticoids and thyroxine [5], which further provides a new perspective on their potential role in hormone-dependent diseases such as breast and prostate cancers. Recently, the CRIP family has attracted much attention for its role in a variety of human diseases. CRIP is associated with a variety of cancers, such as breast [13], colorectal [12], and gastric cancers [10], and its abnormal expression affects tumor proliferation and metastasis. CRIP2 is closely associated with cardiovascular and inflammatory diseases [28]. The research history of the CRIP family has evolved from the characterization of its domain to understanding its role in physiological and pathological settings. Despite remarkable progress, many unanswered questions still exist about how CRIP proteins precisely regulate cellular function and their potential as therapeutic targets. We present a timeline of important events in the study of CRIP family proteins, from early protein discovery to current functional regulation and disease relevance (Fig. 2).

## The CRIP family: metal-binding properties

CRIP family proteins generally possess LIM domains, a feature that determines their unique metal-binding ability, especially their high affinity for zinc ions (Zn2+). Zinc ions play a key role in maintaining the secondary structure and tertiary folding of CRIP [29]. Homology and phylogenetic analyses suggest that mammalian CRIP2 retains histidine residues near the two Zn2+ binding sites (*CX2CHX2C*), which may be related to the competitive binding of Cu+ to Zn2+ [9]. In addition, studies have shown that CRIP family proteins are homologous to ferredoxin and may be involved in iron metabolism [7], however, no study has clarified their direct binding to ferrous ions., and CRIP protein does not contain iron-sulfur cluster domain of ferredoxin. CRIP family proteins may play an important role in the synergizing regulation of related physiological activities by binding to metal ions, thereby maintaining the homeostasis of the cellular microenvironment. Further systematic evaluation and validation of the potential affinity of CRIP for other metal ions will be helpful for the elucidation of biological functions. In recent years, the link between the CRIP family and metal ion homeostasis disorders has been gradually revealed, and it is considered to be closely related to a variety of pathological states. Cancer cells accumulate copper, and studies have shown that CRIP2 can bind to the antioxidant 1 copper chaperone (ATOX1). The ubiquitination and degradation induced by ATOX1 after transferring copper to CRIP2 can activate autophagy by increasing the reactive oxygen species (ROS) level in lung cancer cells and participating in tumor progression [23]. Verdejo-Torres et al. showed that CRIP2 is a copper response regulator in skeletal muscle cells that can bind copper, and the impaired skeletal muscle differentiation caused by the loss of CRIP2 may be related to the accumulation of copper. CRIP2-deficient differentiated cells showed a significant reduction in the level of metallothionein 1 but no change in the level of metallo-regulatory transcription factor 1, supporting the role of CRIP2 as an indirect transcriptional promoter in metallothionein regulation [9]. These results suggest that CRIP family binding metal properties have profound effects on cell differentiation and metabolism.

CRIP family and metal chaperone proteins have the property of metal binding, but whether the CRIP family belongs to the classical metal chaperone family is still controversial. As important accessory proteins for assembling metalloprotein active centers, metallochaperones play key roles in many biological processes, including respiration and defense against toxic agents [30]. Metal chaperones mainly focus on the transient binding of metal ions and deliver metal to specific enzymes or target proteins through protein-protein interactions. For example, the copper chaperone ATOX1 acts as a “molecular transporter” during the transport of Cu+ [31]. Unlike metal chaperones which play a central role in the active transfer of metal ions, current research on the CRIP family mainly focuses on the binding and local regulation of metal ions; Although the conserved cysteine residues in CRIP form a tetrahedral coordination structure with Zn2+ through thiol groups similar to the classical zinc chaperones [29]. This evidence for protein-protein interactions of CRIP family proteins is however insufficient to support the idea that CRIP family proteins act as metal chaperones. Whether CRIP2 and ATOX1 can competitively bind copper, affect cell biological processes, or accumulate toxic ions remains a focus for future research, and the potential relationship between CRIP2 and metal-dependent cell death (such as copper death) is yet to be further explored. The characteristics of CRIP3 binding to metals also require further experimental exploration.

## Diverse biological functions of the CRIP family

**Fig. 3:**
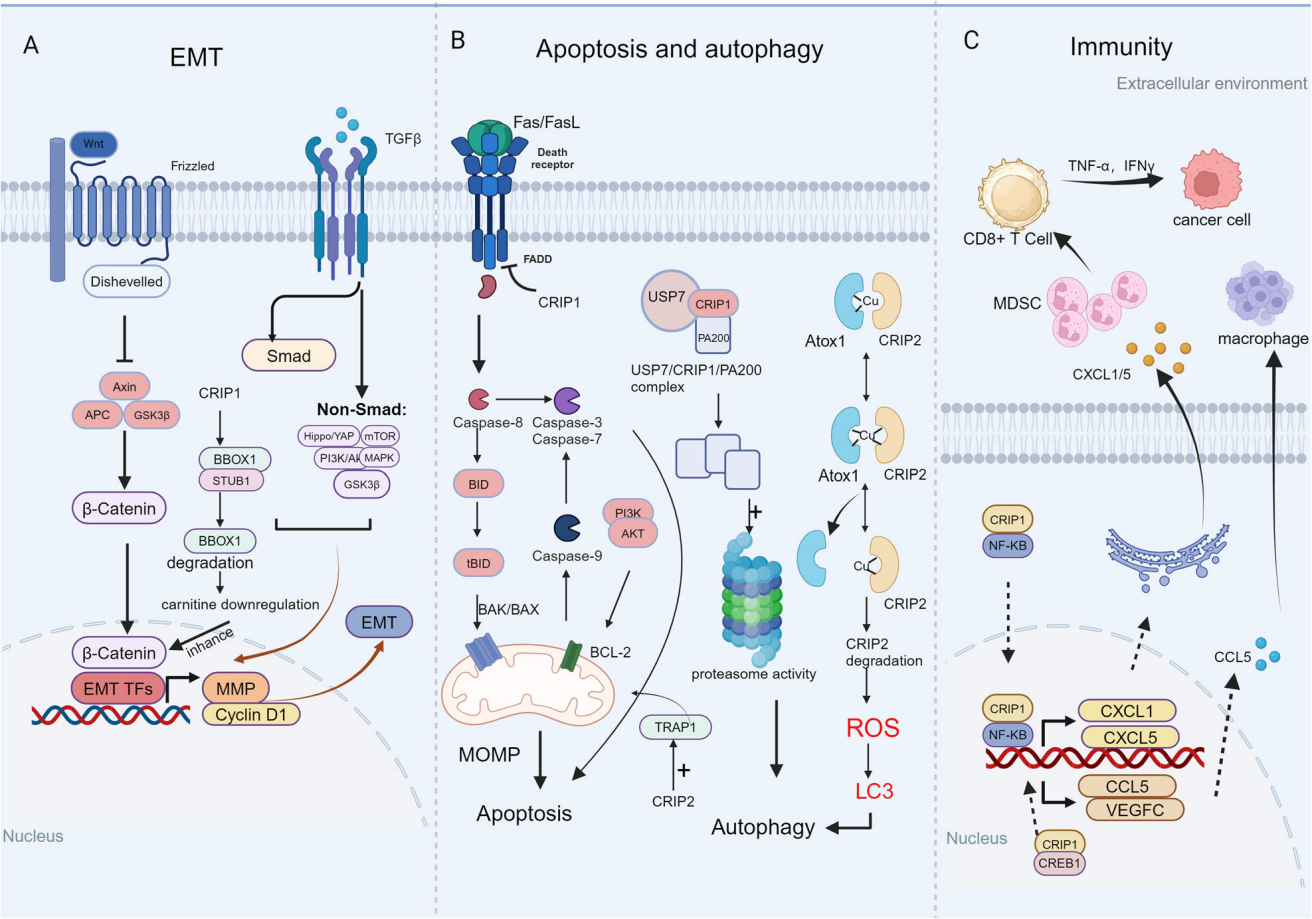
Diverse biological functions of the CRIP family. **A** The CRIP family and epithelial-mesenchymal transition (EMT). CRIP1 induces EMT by activating the Wnt/β-catenin signaling pathway and the GSK 3/mTOR signaling pathway. Additionally, CRIP1 interacts with BBOX1 and STUB1 to promote the ubiquitination and degradation of BBOX1, leading to nuclear accumulation of β-catenin that facilitates EMT induction. **B** The CRIP family and cell death. CRIP1 enhances Fas ubiquitination and degradation, inhibits caspase activation, and suppresses cellular apoptosis. CRIP1 inhibits apoptosis through modulation of the PI3K-Akt pathway. CRIP2 promotes TRAP1 expression to regulate cell apoptosis via mitochondrial pathways. Moreover, CRIP1 increases proteasome activity and autophagy through the CRIP1/USP7/PA200 axis. CRIP2 interacts with ATOX1 and ATOX1 transfers copper to CRIP2 causing ubiquitination and degradation of CRIP2 to increase ROS levels and activate autophagy. **C** The CRIP family and immunity. CRIP1 binds to NF-κB promoting its nuclear translocation while transcriptionally activating CXCL1/5 and promotes chemotactic migration and recruitment of MDSCs. Additionally, CRIP1 interacts with CREB1 to promote the expression of CCL5, recruit macrophages to promote the secretion of TNF-α, and finally enhance lymphatic permeability to cause tumor cell migration.

To describe the diverse biological functions of CRIP family, we used a graphic to show the specific mechanisms of CRIP family involved in EMT, cell death and immunity (Fig. 3).

### CRIP family and epithelial-mesenchymal transition

The phenomenon of epithelial cells acquiring mesenchymal properties, called epithelial-mesenchymal transition (EMT), has been observed in physiological and pathological processes, involving embryogenesis, inflammation, fibrosis, wound healing, and cancer progression [32]. CRIP1 is thought to be involved in epicardial EMT in heart development [33]. Under normal circumstances, epicardial cells can fill the subepicardial space through EMT, and complete the transformation into fibroblasts and smooth muscle cells. Streef et al. found that CRIP1 was highly expressed in the epithelial state, whereas its expression decreased after epithelial-mesenchymal differentiation of transforming growth factor β was induced [33]. In cell culture, CRIP1 knockdown was also observed to induce EMT rapidly. As an important process of tumor invasion and metastasis, abnormal activation of EMT can promote migration and invasion, increase tumor stemness, and enhance drug resistance to chemotherapy and immunotherapy [34]. Studies have found that CRIP1 knockdown increases the invasive potential of breast cancer cells in vitro [13], promotes cell proliferation, and indicates a good prognosis when highly expressed. However, in other tumors, the opposite is observed. In multiple myeloma, the high expression of CRIP1 promotes cell proliferation and invasion, which is an independent risk factor for prognosis [35]. Overexpression of CRIP1 in hepatocellular carcinoma (HCC) promotes the invasion ability of HCC cells and induces EMT [36]. In serous epithelial ovarian cancer, CRIP1 knockdown can also induce the downregulation of EMT marker N-cadherin and inhibit cell migration and invasion [37]. In cervical cancer, compared with adjacent paracancerous tissues, cancer tissues have higher CRIP1 expression, which promotes the occurrence of EMT [38].

Current studies have shown that CRIP1-induced EMT signaling pathways may involve the activation of the Wnt/β-catenin signaling, GSK 3/mTOR signaling, and non-canonical Wnt pathways. In HCC, studies have found that CRIP1 overexpression promotes EMT of liver cancer cells by activating the Wnt/β-catenin signaling pathway [36]. Activation of similar signaling pathways has also been observed in ovarian and cervical cancers. Wnt receptor-ligand interactions can enhance the accumulation and stability of β-catenin in the cytoplasm, thereby stimulating EMT and targeting gene expression upon nuclear translocation [39], thus, affecting cell proliferation, invasion, and migration. This also explains that tumor cells with high expression of Wnt are more likely to undergo EMT and have greater migration and invasion potential. Conversely, in colorectal cancer cells, excessive Zn2+ supplementation activates the GSK 3/mTOR signaling pathway and promotes EMT, CRIP1 silencing inhibits this signaling pathway and reverses this response [40]. EMT is a complex biological process regulated by multiple signaling pathways, and the Wnt/β-catenin signaling pathway is highly interactive with the TGF-β or Notch signaling pathway [41]. Exploring the role of CRIP1 and other key pathways of EMT can provide insights into the differences of CRIP1 in diverse tumors.

In CRIP2 and CRIP3, the highly conserved amino acid sequences result in their great exploration space and potential in EMT. Unfortunately, no direct evidence exists on the association of CRIP2 and CRIP3 with EMT. In summary, although the CRIP family can regulate EMT, they mainly focus on certain cancer types (such as breast cancer and HCC), lack extensive studies on other cancer types, and mostly rely on the detection of changes in EMT markers while ignoring its dynamic process. In the future, we can improve in vivo models and multi-pathway research, find clinical sample support, and transition from basic research to clinical treatment.

### CRIP family and cell death

Cell death, including apoptosis, necroptosis, copper death, and iron death, is a physiological process that maintains the homeostasis of biological development and the internal environment. Autophagy and apoptosis are considered programmed cell death and are genetically regulated under normal physiological conditions [42]. CRIP1 has been confirmed to be involved in activating autophagy and inhibiting apoptosis. CRIP1 promotes its ubiquitination and degradation by interacting with Fas and inhibits the formation of signal transduction complexes and subsequent caspase activation, thereby inhibiting cell apoptosis in colorectal cancer [43]. CRIP1 enhances proteasome activity and autophagy by binding to the ubiquitin specific peptidase 7 (USP7) and the proteasome activating protein 200 (PA200) in multiple myeloma [35]. CRIP1 silencing downregulated cleaved caspase 3 and cleaved poly (ADP-ribose) polymerase to activate the mitochondrial apoptosis pathway in HCC cells [36]. Alternatively, Akt phosphorylation was increased after CRIP1 knockdown, which may lead to a reduction in anti-apoptotic signaling. These results suggest that CRIP1 may inhibit PI3K-Akt pathway to inhibit apoptosis [13]. In thyroid cancer [44], CRIP1 silencing cells undergo G1 phase arrest and apoptosis, thereby inhibiting cell proliferation and migration. CRIP1 silencing in acute myeloid leukemia (AML) induces apoptosis and growth cycle arrest [45]. CRIP1 in human skin fibroblasts inhibits cell proliferation and protects cells from stress-induced death [12]. Therefore, CRIP1 may inhibit cell death to promote tumor growth, but the specific mechanism remains unclear, and the functional heterogeneity of CRIP1 between cells in different tissues has not been well explained. The exploration of the correlation between CRIP1 expression and the survival and prognosis of patients with cancer will further confirm the feasibility of CRIP1 as an intervention target.

*CRIP2* is considered to be a mitophagy-related gene in multiple myeloma, which can be used to predict the survival rate of multiple myeloma, and high CRIP2 expression is usually associated with poor prognosis [46]. In classical literature, CRIP2 was identified as a nuclear copper-binding protein and an autophagy inhibitory protein [23]. CRIP2 interacts with ATOX1, and ATOX1 transfers copper to CRIP2 causing its ubiquitination and degradation to increase ROS levels and activate autophagy. This important finding reveals a potential mechanism by which copper-induced CRIP2 degradation affects autophagy activation. In addition, Bodaar et al. demonstrated that polycomb repressive complex 2 (PRC2) induced tumor necrosis factor (TNF) receptor-associated protein 1 (TRAP1) expression and mitochondrial apoptotic resistance through CRIP2 [47]. CRIP2 overexpression further induces apoptosis by inducing the activation of caspase 3 and caspase 9 proteins [48]. Although there are few studies on CRIP2 and cell death, existing research has revealed a new function of CRIP2 as a nuclear copper-binding protein involved in protein-protein interactions and also enriched the correlation between metal metabolism and cell death by linking copper metabolism to autophagy regulation. It provides a new perspective and inspiration for the CRIP family and metal-dependent death. Although the direct association of CRIP3 with programmed cell death has not been revealed, its conserved domain may provide key clues for research in this field.

### CRIP family and immunization

Immunity is a physiological function of the human body, which can destroy and reject non-self components, and maintain the physiological balance of the body. CRIP family plays an important role in the body’s immunity. CRIP1 is highly expressed in intestinal tracts and immune cells, especially in the cytoplasm of intestinal Paneth cells. Paneth cells are characteristic cells of the small intestinal gland and have a killing effect on intestinal microbes, and the localization of CRIP1 in Paneth cells and monocytes suggests that CRIP1 may be involved in host defense mechanisms and tissue differentiation processes common to these cell types [49]. CRIP1 levels in peritoneal macrophages, spleen, and intestinal tract increase when rats are challenged with lipopolysaccharide [11], suggesting that CRIP1 may play a role in the acute immune response period. In transgenic mouse experiments, flow cytometry data showed that CRIP1 overexpression mice had more CD4+/CD8+ thymic lymphocytes, and CRIP1 expression affected serum concentrations of interferon-γ, TNF-α, and cytokine interleukin [6], indicating that CRIP1 affected cytokine production. It may drive T-cell differentiation and play a role in cellular pathways involved in Th1/Th2 cytokine balance. It was also found that CRIP1 overexpression enhanced interleukin (IL)-6 promoter activity, regulating the cytokine to play a role in immune response [6]. Single-cell sequencing analysis showed that CRIP1 levels were increased in most patient cell types with non-surgical periodontitis [50]. This evidence suggests that CRIP1 may be involved in regulating immune cell differentiation and activation, stimulating the expression of inflammatory factors, and participating in inflammatory response.

In the pathogenesis of human hypertension, angiotensin II upregulates CRIP1 expression in splenic monocytes/macrophages and blood monocytes. CRIP1 may be involved in the development of hypertension through the immune system, especially monocytes [51]. In addition to hypertension, the CRIP family has been reported to affect tumor immune responses possibly through immune escape mechanisms. Bioinformatics studies have shown that CRIP1 is upregulated in AML and exhausted CD8+ T-cells, and high expression indicates a poor prognosis of AML [52]. The increase in CRIP1 level is associated with prognosis and immune cell infiltration in patients with ovarian cancer and sarcoma [53, 54]. CRIP1 is negatively correlated with Th2 and natural killer cells in patients with ovarian cancer, suggesting that patients with tumors having high CRIP1 expression may have immunosuppression; gene enrichment shows that it may be related to abnormal activation of JAK/STAT signaling pathway. However, the increase of CRIP1 level in sarcoma patients is very important for predicting the good outcome of patients, and the infiltration of activated B cells, NK cells and Th1 cells in the immune microenvironment is also related to the improvement of prognosis, suggesting that sarcoma patients with high CRIP1 expression may enhance the anti-tumor immune response of tumors. The results of pancreatic ductal adenocarcinoma showed that the tumor cells with high CRIP1 expression had a high degree of infiltration of myeloid-derived suppressor cells(MDSC) and formed an immunosuppressive tumor microenvironment [55]. Mechanistically, CRIP1 binds to nuclear factor kappa-B (NF-κB) to promote its nuclear translocation, leading to transcriptional activation of C-X-C motif chemokine ligand 1/5(*CXCL1*/5). *CXCL1/5* activation promotes the migration and recruitment of MDSCs [55]. In gastric cancer, CRIP1 interacts with cAMP response element binding protein 1 for transcription to promote the expression of C-C motif chemokine ligand 5 (*CCL5*), recruit macrophages to promote the secretion of TNF-α, and finally enhance lymphatic permeability to cause tumor cell migration [10]. In addition, CRIP1 can promote proteasome inhibitor resistance through the CRIP1/USP7/PA200 axis [35], which can inhibit the sensitivity of tumor cells to chemotherapeutic drugs such as fluorouracil and gemcitabine to some extent, thus becoming a potential target for immunotherapy.

Bioinformatics analysis showed that *CRIP2* was a biomarker related to the immune microenvironment and a risk gene for the survival of patients with colon cancer. The constructed risk score model of immune-related genes could effectively predict the survival period, and high expression was associated with a lower survival probability [56]. CRIP3 has been confirmed to be a thymus-specific LIM protein. Targeted disruption of CRIP3 protein can reduce the number of thymocytes and may also lead to the imbalance of thymocyte subsets [26]. This study provides a new perspective to further understand the regulatory mechanism of thymic functions; however, their role in specific cell types and whether the specific function of CRIP3 differs during early or later stages of development is unclear. The role of the CRIP family in immune-related pathways may provide potential targets for the treatment of related immune diseases, such as thymic dysgenesis, immunodeficiency diseases, and inflammatory bowel diseases. Elucidation of molecular mechanisms and validation of clinical samples are necessary.

## CRIP family and disease

### CRIP family and cardiovascular disease

Human cohort studies have found that the expression of CRIP1 in splenic monocytes/macrophages and circulating monocytes is significantly affected by angiotensin II and is closely related to the pathogenesis of hypertension [51]. Whole transcriptome analysis confirmed that the expression level of CRIP1 was strongly correlated with hypertension at the population level, and the increase of CRIP1 levels in the blood circulation of patients with hypertension was accompanied by an increased risk of stroke [57]. This study incorporated a large amount of global gene expression data related to hypertension and analyzed different cell types (including monocytes and whole blood), which fully illustrated the great potential of CRIP1 as a biomarker associated with hypertension and long-term blood pressure control. However, the specific functions and mechanisms underlying this association were not elucidated. CRIP2, also known as cardiac LIM protein, has been identified as a cardiac vascular marker because its expression has been detected in cardiac endothelial cells during development and in the adult heart [58]. Under normal conditions, CRIP2 changes the expression of extracellular matrix components in the endocardial cushion by inhibiting genes involved in hyaluronic acid synthesis to promote the development of atrioventricular valves [59]. In addition to its fundamental function in the cardiovascular system, CRIP2 is also involved in the pathological changes of cardiovascular diseases. RNA sequencing showed that *CRIP2* is a potentially related gene for myocardial infarction, possibly associated with inflammatory response, NF-κB signaling pathway, and TNF signaling pathway [28]. Studies have shown that CRIP2 is significantly downregulated in mouse models of heart failure and cardiac hypertrophy. Its overexpression reduces Ang II-induced cardiomyocyte hypertrophy, and CRIP2 may play a corresponding role through cardiac remodeling [15]. CRIP2 plays an important role in cardiovascular development, but unfortunately, the existing studies lack systematic molecular mechanism analysis and large-scale clinical data support. As a member of the CRIP family, the long-term average analysis of blood pressure measurements showed a genetic association between CRIP3 and pulse pressure, and single nucleotide polymorphisms near CRIP3 were associated with pulse pressure [60]. And in patients undergoing surgical repair of tetralogy of Fallot, CRIP3 is upregulated with hyperoxia/standard therapy compared with controlled reoxygenation cardiopulmonary bypass [61]. Therefore, we speculate that the CRIP family can effectively predict the risk of cardiovascular disease, while also having the potential to become an important part of the treatment of cardiovascular disease.

Having shown the important role of the CRIP family in cardiovascular development and cardiovascular disease, we summarize the expression, study samples, and corresponding models of different CRIP family members in various cardiovascular diseases (Table 1).

**Table 1:** CRIP family related cardiovascular disease and expression.

CRIP members	Associated diseases	Expression level	Sample	Disease models or patients	References
CRIP1	Hypertension	↑	Splenic monocytes/macrophages and circulating monocytes	Human cohort study and hypertensive model (mouse)	[51]
CRIP1	Hypertension, cardiac hypertrophy, stroke	↑	Whole blood and monocyte	Patients with hypertension and atherosclerosis	[57]
CRIP1	Cerebral ischemic injury	↑	Neurons	Middle cerebral artery occlusion model(rats)	[75]
CRIP2	Pathological cardiac hypertrophy	↓	H9c2	Cardiac hypertrophy model and heart failure model(mice)	[15]
CRIP2	Myocardial infarction	↓	Peripheral Blood	Patients with myocardial infarction	[28]
CRIP2	Cardiac dysfunction	↓	Zebrafish embryonic heart	/	[59]
CRIP3	Hypertension	/	/	Patients with hypertension and atherosclerosis	[60]
CRIP3	Tetralogy of Fallot with hyperoxic/standard vs. controlled reoxygenation cardiopulmonary bypass	CRIP3 is upregulated in hyperoxia/standard compared to controlled reoxygenation cardiopulmonary bypass	Ventricular myocardial biopsy	Cyanotic patients undergoing repair of heart defects	[61]

### CRIP family and cancer

In recent years, scholars have studied the relationship between the CRIP family and tumors. Despite accumulating evidence for the dual function of *CRIP* as oncogenes or tumor suppressors and their associated signaling pathways in specific cancer types, it remains unclear under which circumstances CRIP acts as tumor suppressors or promoters. In most solid tumors, CRIP1 is thought to be highly expressed and promotes tumor progression. For example, in prostate cancer, CRIP1 upregulation increases orthotopic prostate tumor growth [62]. In colorectal [12] and thyroid cancers [44], CRIP1 silencing significantly inhibits cell proliferation, migration, and invasion. Similar results have been observed in gastric cancer [10], cervical cancer [38], HCC [36], serous epithelial ovarian cancer [37], and AML [45]. On the contrary, scholars believe that *CRIP1* acts as a tumor suppressor gene. In breast cancer and osteosarcoma [13, 63], reducing CRIP1 levels may increase cell proliferation, activate cell growth, increase invasion in vitro, and be associated with a worse prognosis. In cutaneous melanoma, CRIP1 expression levels are lower than in normal tissues and are associated with poor prognosis, inhibiting the proliferation, migration and invasion of melanoma cells in vitro [64]. Studies in esophageal squamous cell carcinoma have found that CRIP1 promoter hypermethylation, and complete silencing of CRIP1 promotes tumor progression [65].

At present, the research on CRIP1 and tumors is abundant, with an increasing annual trend, providing more evidence and mechanisms to confirm. There is evidence that CRIP1 promotes the migration of tumor cells to different organs by activating Wnt/β-catenin signaling pathway [36]. In HCC, CRIP1 interacts with gamma-butyrobetaine hydroxylase 1(BBOX1) and E3 ligase STIP1 homology and U-box containing protein 1 (STUB1) to promote BBOX1 ubiquitination and proteasome degradation, thereby reducing the acetylation of β-catenin, promoting its nuclear accumulation, and promoting the proliferation and differentiation of cancer cells, which is clinically associated with a worse prognosis [4]. In addition, CRIP1 can also activate JAK/STAT [53], TNFα-Nfκb [66], Ras/Raf/MEK/ERK, PI3K/AKT [67], and GSK 3/mTOR signaling pathways [40] to achieve the above functions. In addition, tumor invasion and metastasis are inseparable from the degradation of extracellular matrix, which is related to matrix metalloproteinase [68]. As a tumor suppressor, CRIP1 is slightly expressed in breast cancer, and matrix metalloproteinase increases after downregulation, showing greater invasive potential. Additionally, increased phosphorylation of mitogen-activated protein kinase, which promotes proliferation, growth, and migration through phosphorylation of other key regulators and transcription factors, is observed after CRIP1 knockdown [13]. However, CRIP1 inhibits the progression of cutaneous melanoma by inhibiting the implementation of mitochondrial function. CRIP1 inhibits the cytosolic encoded mitochondrial transcription factor A, thereby inhibiting the relative mitochondrial content, mitochondrial DNA copy number, ATP production, respiratory capacity, and the expression level of oxidative phosphorylation related proteins [64]. Therefore, we hypothesized that the different tumor properties exhibited by CRIP1 were associated with the activation of different signaling pathways.

Interestingly, CRIP2 is more likely to act as a tumor suppressor. Bioinformatics analysis showed that low expression of CRIP2 was associated with later tumor stage and poorer prognosis in esophageal cancer [69]. CRIP2 suppresses HIF-1α-mediated glycolysis by interacting with homeobox protein A9, thereby inhibiting the progression of cutaneous squamous cell carcinoma [70]. Studies have shown that CRIP2 can reduce cell growth and invasion and inhibit the progression of esophageal squamous cell carcinoma, which may be related to cell apoptosis induced by changes in caspase activity [48]. Similarly, CRIP2 inhibits tumor progression and angiogenesis by inhibiting NF-κB-mediated transcription of pro-angiogenic cytokines IL-6, IL-8, and vascular endothelial growth factor [71], providing important evidence that CRIP2 acts as a tumor suppressor. CRIP3, as the least studied molecule in the CRIP family, remains the focus of some bioinformatics analysis. Studies have shown that CRIP3 is also closely related to tumorigenesis and can be used as a biomarker of epithelial tumors such as head and neck squamous cell carcinoma [72], lung squamous cell carcinoma [73], and prostate cancer [74], and constructed models can effectively predict the prognosis. However, its oncogenic or tumor-suppressive effects are still unknown.

The function of the CRIP family, as expressed in tumors, also shows tumor type-specific characteristics, which may be related to the characteristics of the tumor itself. The synergistic or antagonistic effects of different CRIP family members in the same tumor type deserve further study. It is believed that with the deepening of research, the clinical application of the CRIP family in tumor diagnosis and treatment will be further clarified. Here, we provide a detailed list of the expression, function and molecular mechanisms of CRIP family members in tumors, so as to facilitate the collection of information for further exploration. (Table 2)

**Table 2:** Expression, function and molecular mechanism of CRIP family proteins in different tumor types.

CRIP members	Expression level	Function	Mechanism	Cancer	Cell line	References
CRIP1	↑	Facilitate cancer stem-like properties	CRIP1/BBOX1/β-catenin	Hepatocellular carcinoma	Hun7, Hep3B, MHCC-97H	[4]
CRIP1	↑	Lymphangiogenesis↑lymphatic permeability↑proliferative and metastatic capacities↑	CRIP1-CREB1 ↑ → VEGFC ↑ CCL5↑	Gastric cancer	MGC-803, HGC-27	[10]
CRIP1	↑	Cell migration and invasion↑	/	Colorectal cancer	SW620,HT29	[12]
CRIP1	Related to HER2	Tumor-suppressive function proliferation and invasion↓	CRIP1 ↓ → phosphorylation of MAPK and Akt↑	Breast cancer	T47D,BT474	[13]
CRIP1	↑	Promote proteasome activity and autophagosome maturation, cell proliferation and invasion↑	CRIP1/USP7/PA200 complex	Multiple myeloma	KMS11,NCI-H929,ARP1	[35]
CRIP1	↑	Cell migration and invasion↑ EMT↑	Activate the Wnt/β-catenin pathway	Epithelial ovarian cancer	A2780,OVCAR3	[37]
CRIP1	↑	Cell migration and invasion↑ EMT↑	Activate the Wnt/β-catenin pathway	Cervical cancer	Hela,Siha,ME180,Caski,MS751	[38]
CRIP1	↑	Cell migration and invasion↑ EMT↑	Activate the GSK 3/mTOR signaling pathway	Colorectal cancer	SW620,LoVo	[40]
CRIP1	↑	Cell proliferation↑	Negatively regulating Fas-related pathway	Colorectal cancer	LS174T,RKO,HT29,HCT116,SW480,SW620	[43]
CRIP1	↑	Proliferation, migration, and invasion↑an independent prognostic marker	Silencing CRIP1 induces apoptosis and G1 arrest	Thyroid carcinoma	K1, TPC-1, TT, SW579	[44]
CRIP1	↑	Cell growth, migration and colony formation↑chemosensitivity to Ara-C↓	Activate the Wnt/β-catenin pathway	Acute myeloid leukemia	U937, THP1	[45]
CRIP1	↑	Proliferation↑	May be involved in the JAK-STAT signaling pathway	Ovarian cancer	A2780, SKOV-3	[53]
CRIP1	Frequently upregulated	Fostered an immunosuppressive tumor microenvironment	CRIP1/NF-κB/CXCL axis	Pancreatic ductal adenocarcinoma	MiaCaPa-2,PANC-1,SW1990,BxPc-3,CFPAC-1	[55]
CRIP1	↑	Cell proliferation, development	Related to FGF-8	Prostate cancer	PC-3	[62]
CRIP1	↓	Inhibition of mitochondrial function	Suppress the protein levels of TFAM	Cutaneous melanoma	SK-MEL-2, A375	[64]
CRIP1	↓	Suppressor of tumor growth	Gene silencing	Esophageal squamous cell carcinoma	KYSE	[65]
CRIP1	↑	Proliferation, migration, and invasion↑	CRIP1/Ras/PI3K/AKT	Hepatocellular carcinoma	HepG2,HCC-LM3	[67]
CRIP1	↓	Tumor proliferation and immune infiltration	/	Nasopharyngeal carcinoma	/	[78]
CRIP1	↑	Possible biomarker and therapeutic target	Hsa-miR-221-5p	Renal cell carcinoma	/	[80]
CRIP1	/	Predict risk of recurrence	DNA methylation	Prostate cancer	/	[82]
CRIP1	↑	Prognosis	/	Acute myeloid leukemia	/	[93]
CRIP2	↑	Mitophagy-related gene	/	Multiple myeloma	/	[46]
CRIP2	/	Mitochondrial apoptosis resistance	PRC2 ↓ → CRIP2 ↑ → TRAP1↑	T-cell acute lymphoblastic leukemia	CCRF-CEM,DND41,PF382,MOLT4,RPMI8402,Jurkat	[47]
CRIP2	↓	Colony formation, growth, and invasion abilities↓	CRIP2 ↑ → caspases 3 and 9 ↑ →apoptosis↑	esophageal squamous cell carcinoma	81T, EC1,EC18,HKESC-2,KYSE,SLMT,TTn-6	[48]
CRIP2	↓	An immune-related dangerous gene	/	Colon cancer	/	[56]
CRIP2	↓	Inhibit glycolysis tumor-suppressive function	miR-365-HOXA9-HIF-1α	Cutaneous squamous cell carcinoma	A431	[70]
CRIP2	↑	Impact the X-ray radiosensitivity of NSCLC	Regulate the occurrence of apoptosis and cell cycle arrest	Non-small cell lung cancer	A549-R11	[84]
CRIP3	/	Prognostic biomarker	/	Squamous cell carcinoma of the head and neck	/	[72]
CRIP3	/	Prognosis	CRIP3 DNA methylation ↓ →poorer prognosis	Lung squamous cell carcinoma	/	[73]
CRIP3	/	Prognosis	Methylation	Prostate cancer	/	[74]

## Clinical application

### CRIP family holds promise as potential disease biomarkers

In recent years, the CRIP family has shown great potential as biomarkers of diseases. In benign diseases, the expression of CRIP1 is upregulated in nerve cells after cerebral ischemia; and is considered to be an important target for preventing cerebral ischemic injury [75]. *CRIP1* is a differentially expressed gene in the synovium and blood of patients with osteoarthritis and has diagnostic value for osteoarthritis [76]. In addition, CRIP1 is also highly expressed in liver cirrhosis, and single-cell transcriptome results show that *CRIP1* is the core driver gene of hepatic stellate cell activation [77]. CRIP2 expression has been reported to be elevated in the serum and cerebrospinal fluid of patients with trigeminal neuralgia; successful microvascular decompression procedures normalize the levels of these proteins, which can be used as biomarkers for effective treatment [16].

In cancer, CRIP can be used as an auxiliary diagnostic indicator, and its gene methylation status can be used as an independent prognostic indicator for a variety of cancers. Bioinformatics analysis showed that CRIP1 could be a potential biomarker to predict the prognosis of nasopharyngeal carcinoma [78]. CRIP1 is downregulated in breast cancer and osteosarcoma, which indicates a higher survival rate and is a marker for predicting good prognosis [13, 63]. *CRIP1* is a key gene in epithelial ovarian cancer. The expression of CRIP1 in tumor tissues is higher than that in adjacent tissues, which is related to higher pathological stage, grade, and lymph node metastasis [37]. Moreover, CRIP1 has prognostic significance in prostate cancer [79], hypertension-related renal cell carcinoma [80], ependymoma [81], etc. Studies have shown that in prostate cancer, the hypomethylation level of the *CRIP1* promoter is associated with poor prognosis [82]. However, in breast cancer, the methylation frequency of CRIP1 increases with the increase of breast cancer tumor stage, and its hypermethylation is associated with poor clinical prognosis [83]. Similarly, CRIP2 has shown great potential in the diagnosis and prediction of diseases such as esophageal cancer [69], non-small cell lung cancer [84], colon cancer [56], high-grade serous ovarian cancer [85], and acute lymphoblastic leukemia [47]. Bioinformatics analysis showed that the low expression of CRIP2 was associated with a later tumor stage of esophageal cancer. CRIP2 expression is upregulated in radiation-resistant non-small cell lung cancer, which may be achieved by regulating cell apoptosis and cell cycle [84]. CRIP3 was identified as a novel prognostic biomarker in cancers such as head and neck squamous cell carcinoma [72], lung squamous cell carcinoma [73], and prostate cancer [74], among which the CRIP3 hypomethylated epigenotype in lung squamous cell carcinoma was significantly associated with poor prognosis. The methylation status of CRIP3 in prostate biopsy and urine is an important marker for postoperative recurrence and prognosis of patients with prostate cancer [74, 86]. In summary, CRIP family members have important potential in the early diagnosis and prognostic evaluation of diseases, as well as in the monitoring of treatment response. In the future, with the deepening of research, the application prospect of the CRIP family as multifunctional biomarkers is worth looking forward to.

### Clinical perspectives of the CRIP family in tumor

CD8+ T-cells are the main executor of anti-tumor immune response, and the reduced activation of CD8+ T-cells up-regulates the expression of programmed cell death protein 1 to promote T-cell exhaustion, destroy the immune homeostasis of T lymphocytes, and induce tumor cell escape [87, 88]. That is, in malignant tumors, CD8+ T-cell infiltration implies a better prognosis. Studies have shown that pancreatic ductal adenocarcinoma is one of the most immunosuppressive tumor types, and the increased expression of CRIP1 activates the CRIP1/NF-κB/CXCL axis to drive the migration and recruitment of MDSC and the infiltration of low CD8+ T-cells to form an immunosuppressive microenvironment [55]. SX-682 is a CXCR1/2 receptor inhibitor that blocks tumor MDSC recruitment and enhances T-cell activation. The combination of anti-programmed cell death ligand 1 (PD-L1) treatment and SX-682 increased CD8+ T-cell infiltration and enhanced anti-tumor activity in CRIP1-overexpressing mice, indicating that CRIP1-involved signaling pathways are critical for tumor immune escape and TME formation. Targeting this signaling pathway enhances the activation of T-cells and makes them more sensitive to immunotherapy. CRIP1 is upregulated in exhausted CD8+ T-cells, and the development of inhibitors against CRIP1 may relieve immunosuppression, improve exhausted CD8^+^ T-cells, and enhance anti-tumor ability. In addition, PD-L1 is an important immune checkpoint. Analyzing the correlation between CRIP1 and PD-L1 expression in different tumors, studying the clinical association between CRIP1 and PD-L1, and combining CRIP1-targeted therapy and anti-PD-L1 therapy may provide new ideas for tumor immunotherapy.

In addition to PD-L1, we explored the potential association between CRIP1 and human epidermal growth factor receptor 2 (HER2), a key breast cancer target. Based on imaging mass spectrometry analysis, Rauser’s study found that CRIP1 and HER2 show co-expression or highly correlated spatial distribution in tissues, and CRIP1 is significantly upregulated in *HER2*-positive breast cancer. CRIP1 may be used as a serum biomarker in patients with breast cancer to assist in molecular typing [89]. The combination of CRIP1-targeted drugs and anti-HER2-targeted therapies, such as trastuzumab, will bring greater clinical benefit to patients with *HER2*-positive breast cancer. In addition, CRIP1 expression can change the expression of interferon-γ, IL-6, and IL-10 [90]. The excessive production and signaling disorder of IL-6 are associated with various cancers. Targeted inhibition of IL-6 signaling pathway by monoclonal antibodies or IL-6Rs can produce therapeutic benefits for a variety of malignant tumors.

The deletion of PRC2 in T-cell acute lymphoblastic leukemia may induce mitochondrial chaperone TRAP1 expression by up-regulating CRIP2, thus achieving mitochondrial apoptosis resistance [47]. CRIP2 is a direct target of PRC2. Upregulation of CRIP2 and activation of the downstream TRAP1 mitochondrial chaperone can act as key mediators of apoptosis resistance. Clinical development of CRIP2 inhibitors may, therefore, provide a new drug-resistant treatment strategy. While the exploration of the physiological functions and tumor-specific mechanisms of CRIP3 in thymus development may also reveal its effectiveness as a target. In summary, the development of molecular inhibitors of the CRIP family has great potential in improving drug resistance, inhibiting tumor cell proliferation and metastasis, and may be used in combination with existing regimens to achieve personalized treatment for patients with cancer; however, the selectivity of targeting is still an important challenge in current research.

## Discussion

CRIP family includes CRIP1, CRIP2 and CRIP3. Given the diverse biological functions of the CRIP family in cell EMT, cell death, and immunity, its role in tumors and cardiovascular diseases is self-evident [35, 37, 52]. CRIP family acts as tumor promoters or suppressors, affecting the development of the cardiovascular system and disease occurrence under physiological and pathological conditions, and may also regulate the inflammatory process by affecting the expression of cytokines [91]. CRIP family is involved in a variety of complex potential regulatory mechanisms, including NF-κB, Wnt/β-catenin, Ras/Raf/MEK/ERK, PI3K/Akt, GSK 3/mTOR and Fas/FasL signaling pathways, which have become potential biomarkers for clinical diagnosis, prognosis and treatment of tumors. It has a good clinical application prospect in tumor immunity.

Although numerous studies have revealed the functions and mechanisms of the CRIP family, there are still some questions to be explored. First, the CRIP family, as an important zinc-binding protein, may induce metal exchange. Studies have shown that CRIP1 preferably binds to zinc ions, whereas CRIP2 also has a high affinity for copper [9, 29]. However, no relevant studies have revealed the mechanism and biological significance of their alternate binding to metals under physiological and pathological conditions. Second, the specific signaling pathways and molecular targets of the CRIP family in different diseases have not been fully revealed. The different CRIP family members may show distinct functions in the same pathological process, thus necessitating more complete mechanism research. In addition, as potential biomarkers for clinical diagnosis, prognosis, and treatment of patients with cancer, the research on CRIP is limited to bioinformatics and cell data, and there is a lack of large-scale, multicenter, and prospective clinical studies. It is necessary to expand the clinical sample size and combine it with patient cohort analysis to further clarify the specificity and sensitivity of the CRIP family as biomarkers.

Increasing evidence has shown that epigenetic regulation plays an important role in tumor growth, immune escape, metabolic reprogramming, and treatment resistance [92, 93]. Studies have shown that CRIP family methylation status can be an independent prognostic indicator for patients with cancer, however, most of the current studies are based on bioinformatics prediction, lacking important experimental basis and regulatory mechanisms. It is of great significance to explore the DNA methylation, protein modification, and non-coding RNA regulation of the CRIP family for enriching the cellular epigenetic regulatory network. In summary, our review reveals the structure, function, and important links between the CRIP family and diseases. The development of inhibitors or agonists will open new avenues for basic and clinical research.

## Data Availability

All data included in this study are available upon request by contacting the corresponding author.
